# Biochemical fingerprint of colorectal cancer cell lines using label‐free live single‐cell Raman spectroscopy

**DOI:** 10.1002/jrs.5389

**Published:** 2018-05-02

**Authors:** Julia Gala de Pablo, Fern J. Armistead, Sally A. Peyman, David Bonthron, Michael Lones, Stephen Smith, Stephen D. Evans

**Affiliations:** ^1^ Molecular and Nanoscale Physics Group, School of Physics and Astronomy University of Leeds Leeds UK; ^2^ Welcome Trust Brenner Building, St James's University Hospital, Faculty of Medicine and Health University of Leeds Leeds UK; ^3^ School of Mathematical and Computer Sciences Heriot‐Watt University Edinburgh UK; ^4^ Department of Electronics University of York York UK

**Keywords:** colorectal cancer, living cells, metastasis, Raman spectroscopy, single cell

## Abstract

Label‐free live single‐cell Raman spectroscopy was used to obtain a chemical fingerprint of colorectal cancer cells including the classification of the SW480 and SW620 cell line model system, derived from primary and secondary tumour cells from the same patient. High‐quality Raman spectra were acquired from hundreds of live cells, showing high reproducibility between experiments. Principal component analysis with linear discriminant analysis yielded the best cell classification, with an accuracy of 98.7 ± 0.3% (standard error) when compared with discrimination trees or support vector machines. SW480 showed higher content of the disordered secondary protein structure Amide III band, whereas SW620 showed larger α‐helix and β‐sheet band content. The SW620 cell line also displayed higher nucleic acid, phosphates, saccharide, and CH_2_ content. HL60, HT29, HCT116, SW620, and SW480 live single‐cell spectra were classified using principal component analysis or linear discriminant analysis with an accuracy of 92.4 ± 0.4% (standard error), showing differences mainly in the β‐sheet content, the cytochrome C bands, the CH‐stretching regions, the lactate contributions, and the DNA content. The lipids contributions above 2,900 cm^−1^ and the lactate contributions at 1,785 cm^−1^ appeared to be dependent on the colorectal adenocarcinoma stage, the advanced stage cell lines showing lower lipid, and higher lactate content. The results demonstrate that these cell lines can be distinguished with high confidence, suggesting that Raman spectroscopy on live cells can distinguish between different disease stages, and could play an important role clinically as a diagnostic tool for cell phenotyping.

## 1 INTRODUCTION

Mapping of tumours, from subcellular to whole organ length scales represents a major challenge in cancer research for understanding how biological changes relate to pathology. Raman spectroscopy probes the vibrational modes of molecules, offering an information‐rich, label‐free technique for studying biological systems. Importantly, the technique can be used to probe living systems, providing biochemical information with subcellular and cellular spatial resolution on live cells.^1^, ^2^, ^3^, ^4^, ^5^ It allows the discrimination between cell types at the single‐cell level and thus has a potential for application in studying cell heterogeneity, differential response to drugs, automatic mapping of tissue samples, and microfluidic‐based identification of cancers.^6^

A number of groups have used Raman in studies on fixed cells, where the proteins within the cells are polymerized, keeping the cells in a non‐viable chemically stable state. However, a number of publications^7^, ^8^, ^9^, ^10^, ^11^, ^12^ and recent reviews^5^, ^13^ indicate that formalin‐fixed cells show a decrease in lipid and protein content, an overall weaker signal, new peaks due to the fixation, and shifts in some bands. Raman spectroscopy has previously been undertaken in live cancer cell lines.^14^, ^15^ A major challenge of Raman spectroscopy in living samples is that it can be complicated by apoptotic effects due to the removal of cell medium, which limits the measurement time, and thus the number of cells typically analysed is often in the low tens.

Cells have a rich spectral content, which provides Raman with great potential as a diagnostic tool.^16^ However, the differences between cell types are usually subtle. This, coupled with the need to sample large numbers of cells, means that multivariate analysis for discrimination between cell types or states is required.^17^ Various chemometric methods have been employed to identify the main spectral variations in preprocessed data.^17^, ^18^ The most common method for dimensionality reduction is principal component analysis (PCA), in conjunction with other multivariate methods such as linear discriminant analysis (LDA)^19^ or cluster analysis.^9^, ^20^ LDA is a supervised multivariate method that looks for the axis that maximizes the between‐class separation while minimizing the within‐class scatter.^17^ Other data mining techniques, such as support vector machines (SVM), genetic algorithms, discrimination trees (DTs), or artificial neural networks can be very powerful for class separation but are more difficult to relate to the underlying biology.^21^ Tree classifiers, also known as DTs, have been less widely used,^22^, ^23^ and even though they are sometimes less powerful than the previously mentioned classifiers, their output is easier to relate to the original spectral features, and they can also capture non‐linear relationships within the data. SVM are very powerful classification methods^24^, ^25^, but it is sometimes difficult to extract useful knowledge from the trained models. Partial least square regression (PLSR) is a dimensionality reduction method alternative to PCA that allows assigning scores to each of the groups, finding the components that correlate with a particular characteristic of the classes.^26^ It has previously been used to correlate metastasis potential with metabolic data.^27^

Colorectal cancer has an estimated mortality of 56% (2012), and around 20% of diagnosed patients already have metastases at diagnosis.^28^ Isolating the chemical fingerprint of metastatic colorectal cells will aid tissue and single‐cell studies on the effectiveness of preoperative treatments and tumour identification. For this study, the main cell lines chosen were SW480 and SW620, derived from a primary Duke's stage B adenocarcinoma and secondary tumour in a lymph node from the same patient.^29^, ^30^, ^31^ Using these cell lines can help isolate metastasis variability from the person‐to‐person variation. Previous reports of vibrational spectroscopy on the SW620 or SW480 model system at the single‐cell level have been undertaken using synchrotron Fourier transform infrared microspectroscopy on live cells^32^ and Raman spectroscopy of a small number of fixed cells combined with stimulated Raman scattering.^33^

In addition, HL60, HCT116, and HT29 cells were analysed. HL60 is a nonadherent blood cell line derived from human promyelocytic leukaemia and was used to show the ability of Raman to differentiate between cell lines with very different origins. HCT116 cells are derived from human colon carcinoma, so are expected to show similarities with primary colon cancer cell lines and will challenge the system to separate between different cancer types from the same tissue. HT29 cells are derived from Duke's C stage human colon adenocarcinoma and are thus expected to show similarities with the SW480 cell line that is human colon adenocarcinoma Duke's stage B, challenging the system to differentiate between different stages of the same disease. A schematic outlining the Duke's stages of colorectal adenocarcinoma is shown in Figure S1. Previous studies have done bulk Raman measurements in HL60 cell pellets,^34^, ^35^, ^36^ on single‐nuclei of HL60 cells,^37^ and on fixed HT29 cells.^38^ Single‐cell live label‐free Raman spectroscopy of these cell lines has been previously done comparing HCT116 cells with HT29 cells,^39^ studying apoptosis induction on HCT116 cells,^40^, ^41^ studying proliferation effects caused by coculture of HL60 cells with mesenchymal stem cells^42^ and comparing HL60 cells with peripheral blood mononuclear cells,^43^ but the number of cells analysed in these studies were always below 30.

Here, we present the first report of Raman spectroscopy on live cells on multiple colorectal cell lines SW480/SW620/HT29/HCT116 and compare these to a noncolorectal cell line such as HL60. Data were obtained from 680 live cells, with excellent reproducibility between experiments. SW480 and SW620 results were first analysed using different multivariate methods—PCA or LDA, DT, and SVM—to find an optimal multivariate method to differentiate between these primary and secondary cancer cells. Then, additional cell lines were added to the analysis to identify possible metastasis biomarkers compared with a greater pool of cells ranging from different disease states, different disease types within the same organ, and different cell origins altogether. Results have successfully classified these cells with high accuracy and identified potential biomarkers that will need to be tested in further experiments in clinical samples.

## 2 MATERIALS AND METHODS

### 2.1 Cell culture

The SW480, SW620, HT29, and HCT116 cell lines were cultured in Dulbecco's Modified Eagle Medium (DMEM/F‐12, Gibco). The HL60 cell line was cultured in Roswell Park Memorial Institute medium (RPMI 1640, Thermo Fischer Scientific). Media were supplemented with 10% fetal bovine serum (Sigma), 2 mM Glutamax (Thermo Fisher Scientific), and penicillin 100 units/ml streptomycin 100 μg/ml (Sigma). Phase contrast images of the SW620 and SW480 cell lines grown in flasks showed a more epithelial‐like morphology for SW480's and a more fibroblast‐like morphology for SW620's as shown in Figure S2. Cells were not “synced” to allow the natural cell cycle within sample variability of the cell lines. All experiments were done with passage numbers below 50. SW480, SW620, HT29, and HCT116 were washed with Dulbecco's phosphate buffered saline (DPBS) and gently retrieved from six‐well plates by incubating with cell dissociation buffer (Thermo Fisher Scientific) for 30 min, followed by centrifugation (100 Gs; 1 min) and resuspension in cell dissociation buffer. HL60 cells were retrieved from media by centrifugation (100 Gs; 1 min) and washed with DPBS once before resuspending in DPBS. When pipetted into the setup, cells sedimented onto the coverslip and showed no visible Brownian motion, remaining in a spherical shape.

### 2.2 Raman spectroscopy

Quartz slides (UQG Optics, 75 × 25 × 1 mm) and coverslips (25.4 × 25.4 × 0.15–0.25 mm Alfa Aesar) were sonicated with acetone (VWR Chemicals), 2–5% Decon 90 (VWR Chemicals) and rinsed with MilliQ. Hydrogen peroxide 30% (Thermo Fischer) and sulfuric acid >95% (Thermo Fischer) were mixed in a 3:7 proportion (Piranha solution) and used to clean the slides for 20 min. Slides and coverslips were stored in MilliQ and dried under a stream of nitrogen immediately before the experiment. Spacers were prepared using a 50 μm polyethylene terephthalate film (Goodfellow, UK). A nitrocellulose‐based solution was used to bond the coverslip to the slide and was dried at 80 °C for 30 min. The cell solution was pipetted into this chamber immediately before measuring. All experiments were done at room temperature and samples were measured for 1 hr.

The Raman system used was an inVia Raman confocal inverted microscope (Renishaw) integrated with a Leica DMi8/SP8 laser scanning confocal microscope system, with a DPSS Diode 532 nm laser (intensity of 22 mW on the sample). Light was collected using a Newton EMCCD Sensor (DU970P, Andor, 1,600 × 200 px). Prior to every experiment, a spectrum of a silicon sample was collected using a 10× objective, and the microscope was calibrated to the peak position (520.5 cm^−1^). The longer‐term aim of our work is to measure the Raman signal of these cells in a microfluidic platform; thus, the Raman spectra of detached cells were measured.

The cell spectra were obtained using a 100× oil objective (HC PL APO CS2 FWD 0.13 mm NA 1.4) and a slit size of 20 μm. This objective and slit opening gave a 10.2 μm full width half maximum confocality when tracking the changes of Raman intensity of the 520.5 cm^−1^ with the distance to a silicon sample, ensuring the whole volume of the cell can be measured when using this configuration. The laser spot was defocused by 50% using a beam expander, generating a laser spot of approximately 20 μm diameter. Each cell spectrum was obtained using a step configuration with 1 s exposure time and five accumulations in two different windows (300–1,800 cm^−1^ and 1,800–3,200 cm^−1^) which gave a total exposure time of 10 s per cell. Between 79 and 85 cell spectra were obtained per experiment, and the data from multiple experiments were combined for this paper (167 SW620 cells, 163 SW480 cells, 89 HL60 cells, 190 HT29 cells, and 71 HCT116 cells) without omitting any outliers. Five background spectra from cell‐free regions of the sample measured at the same Z position as the cells were obtained for each experiment.

### 2.3 Preprocessing of the spectra

The spectra obtained were cosmic ray filtered (WiRE software) and exported as text files for further analysis using Matlab's Statistics and Machine Learning Toolbox (MathWorks). The Matlab functions used are indicated by italics. The silicon peak of a calibration sample was used to calibrate the wavenumber axis of each spectrum and the spectra were translated vertically, such that the minimum intensity was zero. For each cell spectrum, the average background spectrum was multiplied by an adjustment factor before being subtracted from the cell spectrum to ensure the quartz band at around 480 cm^−1^ was fully corrected. The spectrum was smoothed using a Savitzky–Golay filter. The spectra were truncated to only consider the regions between 730 and 3,100 cm^−1^. The spectra were baseline corrected using the algorithm developed by Koch et al. (2016). 44 The regions of the spectra between 1,750 and 2,800 cm^−1^ were not considered for subsequent analysis. In order to normalize to the protein content, for comparison with biochemical literature data, each spectrum was normalized such that the Amide I peak was unity.

### 2.4 Statistical analysis and classification

**Statistical errors:** Unless stated otherwise, all values are expressed ± the standard error calculated as *σ*/ √ *N*, where *σ* is the standard deviation and *N* the sample size. Performance of the multivariate models was calculated as the accuracy of the model using a 10‐fold cross validation with five repetitions. **Correlation matrix:** The correlation matrix of all the preprocessed data was calculated to help with the peak assignment. The function used was *corrcoef*. To simplify the correlation image, point with *p* values >.0001 were set to zero, and only the peaks that showed an absolute value of correlation greater than 0.3 were considered in the analysis. **PCA:** The edited data was truncated to 730–1,750 cm^−1^ and 2,800–3,000 cm^−1^ and standardized using standard normal variate. The function used was *pca*. LDA was performed keeping only the first 25 PCs using the function *fitcdiscr* using a “linear” discriminant type. **DT:** The function used was *fitctree* using the *exact* algorithm that fits a binary classification tree to the data. **C5.0:** R's *C5.0* package was used to train DT ensembles based on R. Quinlan algorithm and the *caret* package was used to optimize training parameters. It trains multiple small DTs and analyses the most frequently chosen wavenumbers. **SVM:** R's *kernlab* package was used to train SVM models, and the *caret* package was used to select an optimal kernel function (from amongst linear, polynomial, and Gaussian kernels). As all the tested kernels showed a similar performance, the linear kernel was selected. **PLSR** function *plsregress* was used for the analysis. Scores in each of the components were compared in pairs using an unpaired two sample one‐tailed *t* tests, and the number of components was determined so cell lines showed a significant (*p* > .01) increase with the adenocarcinoma stage. Final considered values all show at least *p* < .001. Duke's stages (B primary, C primary, C metastasis) were fitted as (1,2,3).

## 3 RESULTS

### 3.1 Distinction between primary and secondary tumour cells

**Figure 1:**
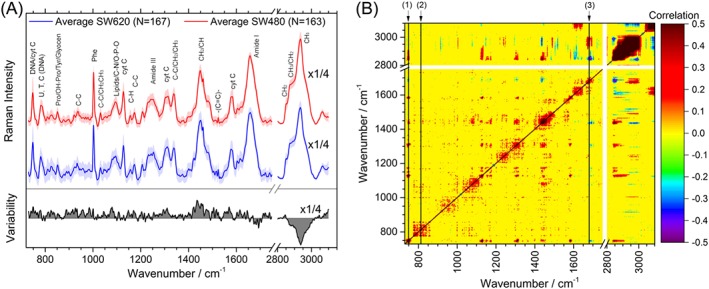
(a) Average single‐cell spectra and variability spectrum, for primary (SW480) and secondary (SW620) cells. The error around the average shows one standard deviation. The region around 2,900 cm^−1^ is shown reduced by a factor of 4 to enhance the details in the fingerprint region. (b) Correlation matrix of the different bands for all cells, where the points with *p* values >10^−4^ were considered not significant and set to 0 to simplify the plot [Colour figure can be viewed at http://wileyonlinelibrary.com]

Figure 1a shows the SW620/SW480 averaged spectra, normalized to the Amide I peak, and variability for each cell line. The main peaks have been identified in accord with the established literature and are given in Table S1.^45^, ^46^, ^47^, ^48^, ^49^, ^50^

The –CH_2_ and –CH_3_ stretching contributions in the region of 2,800–3,200 cm^−1^ showed higher overall intensity for SW480 cells and a greater CH_2_: CH_3_ ratio for SW620 cells, indicating differences in lipid composition between the two cell lines with higher lipid content for the larger size cells SW480 (SW480 diameter = 16.9 ± 0.4 μm cf. SW620 diameter = 14.4 ± 0.3 μm) and in agreement with previous reports on fixed SW480/SW620 cells.^33^ The fits of these peaks are shown in Figure S3a and S3b.

The Amide III band (1,230–1,300 cm^−1^) and the Amide I band (1,600–1,690 cm^−1^) are widely used for studying the protein secondary structure. Peaks fitted to the Amide III β‐sheet, α‐helix, α + β, and disordered structures showed that disordered structure was higher for the SW480 cells, and β‐sheet was higher for SW620 cells, whereas the ratios of α:β indicated that SW620 cells had more α‐helix to β‐sheet content ratio than SW480 as shown in Figure S3b1 and S3b2. Other protein related peaks associated with hydroxyproline, proline, and phenylalanine all showed higher intensity for the SW620 cells. The Amide I bands showed a similar trend to the Amide III fitting for the variation in the β‐sheet and α‐helix content.

The 782 cm^−1^ nucleic acid peaks and the 810 cm^−1^ peak usually associated with bonded phosphates or phosphodiester bonds showed a larger contribution for SW620 than of SW480 cells, indicating higher nucleic acid to protein ratio. The 1,338 cm^−1^ band with mixed contributions of DNA and CH vibrations showed this same trend. This is consistent with the SW620 having larger RNA content^51^ and nuclear area^52^ that SW480.

Most of the peaks associated with saccharide contributions show higher contribution in the SW620 spectra. This could be explained by higher concentrations of glycolysis intermediates such as acetate or lactate^53^ and an increased secretion of pericellular hyaluronan in SW620 compared with SW480 cells.^54^ Peaks associated with phosphates also show a higher contribution for the SW620, which is in agreement with previous reports that showed an increase of the phosphorylated status of these cells.^32^ Peaks at around 1,128; 1,310; and 1,585 cm^−1^ have previously been labelled as cytochrome C resonance^46^, ^55^ and can be used to monitor early signs of apoptosis.^46^ Peaks at 1,157; 1,517; 1,525; and 1,620 cm^−1^ reveal higher contributions of double bonds to the SW620 normalized spectra^45^ and have previously been reported as cancer biomarkers in different biological samples, assigning them to carotenoids or porphyrins.^15^, ^33^, ^56^

In summary, when normalizing to the Amide I band, SW620 cells show a larger contribution of α‐helix proteins, saccharides, nucleic acids, and double bonds related bands, whereas SW480 cells show larger contribution of lipids, β‐sheet, and disordered structure proteins.

### 3.2 Peak correlation

To aid peak assignments and help track cell state, we used the *p*‐value filtered correlation matrix of the preprocessed data (Figure 1b). Only correlations with an absolute value higher than 0.3 were considered for this analysis. A series of strongly correlated peaks associated with cytochrome C were found at 748; 1,128; 1,156; 1,175; 1,310; 1,431; 1,438; 1,448; 1,585; 2,845–67 cm^−1^ which had a strong negative correlation with the Amide I peaks at 1,682 and 1,690 cm^−1^ (see 1 in Figure 1b).

Other highly correlated peaks in the spectra are the 810 cm^−1^ series (see 2 in Figure 1b) that positively correlates with 781; 828; and 1,732 cm^−1^. The 810 cm^−1^ is usually labelled as being due to phosphodiester or phosphate vibrations, with the 781 cm^−1^ peak associated with the pyrimidine bases ring breathing mode and the 828 cm^−1^ peak due to phosphates. Overall, this indicates that this series is related to nucleic acid vibrations.

Another notable correlation found is the series of 1,679 cm^−1^ (see 3 in Figure 1b), which shows positive correlation along the Amide I peaks at 1,642; 1,671; 1,687; 1,689; and 1,697 cm^−1^. These bands are related to Amide I β (1,679 and 1,671), α (1,642), and disordered (1,687) structures that all show high correlation.

### 3.3 Primary and secondary cell lines discrimination using classification algorithms

The individual cell spectra were used to classify cells by three different methods: with PCA analysis, with DT—both using an individual DT and using the C5.0 algorithm^57^—and with linear kernel SVM. First, we consider this for the potentially more challenging case of SW480 and SW620 cell lines, which are of the same genetic origin and grown under the same conditions. Once optimized, we then extended this to other cell lines.

#### 3.3.1 Nonsupervised multivariate analysis: PCA

**Figure 2:**
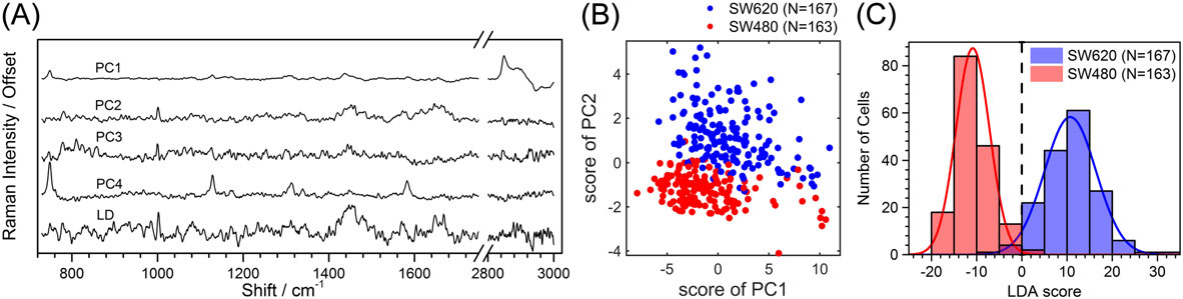
PCA/LDA results. (a) Shape of the PCs 1 to 4 and of the LD (b) 2D plot of the scores for the first two PCs. (c) Histogram of the individual cell scores when projecting the cell data onto the LD from (a) with a vertical dashed line at the point of best separation. LD = linear discriminant; LDA = linear discriminant analysis; PC = principal component; PCA = principal component analysis [Colour figure can be viewed at http://wileyonlinelibrary.com]

Figure 2a gives the first four principal components. Using PC1–3 was enough to separate the two cell lines, by plotting the scores of the first two PCs (Figure 2b). PC1 showed mainly lipid‐related contributions and accounted for 26% of the variability. PC2 and 3 showed mixed RNA and protein‐related contributions and contributed to 5.1% and 3.3% of the variability, respectively. PC4 showed a strong contribution of the cytochrome C resonance. PCA showed that the SW620 cells are more heterogeneous than the SW480 cells, indicating greater within‐class variability. PC2 was the component that better separated the two cell lines and showed two sharp peaks at 781 (DNA) and 1,001 cm^−1^ (phenylalanine) and broader peaks around 1,455 (CH_2_ vibrations); 1,573 (carboxylic group or nucleic acids); and 1,647 cm^−1^ (Amide I). This component seems to be accounting for mixed contributions to proteins, lipids, and nucleic acids. Interestingly, PC4 did not show different contributions between the SW620 and SW480 cells but seemed to be related to the within‐class heterogeneity of the cells. The histograms of the scores are given in Figure S5.

#### 3.3.2 Supervised multivariate analysis: PCA and LDA

Figure 2a shows an example of a linear discriminant (LD) that provides a good classification of the two cell lines (98.7 ± 0.3%). This LD is dominated by the PC2 contribution and shows positive values for SW620 cells and negative values for SW480 cells. The shape of the LD shows the enrichment in CH_2_ ν_s_ of the SW620 and the increased contents in CH_3_ stretching vibrations of SW480 cells. The cytochrome‐associated peaks are absent, indicating that the viability of the cells was similar and that the differences found here are not artefacts due to apoptosis. Modes related to phosphates were in general of negative sign, whereas the amino acid‐related peaks like phenylalanine, tyrosine or hydroxyproline, and the Amide III band show positive contributions. In summary, the LDA/PCA confirm that the SW620 cells have a higher CH_2_:CH_3_ ratio as well as larger contributions from amino acids, phosphates, and proteins than the SW480 cells at a single‐cell level and that these are good biomarkers to classify the cells. The scores for the LD are shown in Figure 2c.

#### 3.3.3 Comparison of performance of different multivariate methods

The performance of all the multivariate methods compared is shown in Figure 4a. The final performance values obtained were of 98.7 ± 0.3% for the PCA/LDA classifier, 86 ± 1% for the simple DT, 94.0 ± 0.9% for the C5.0 DT, and 98.1 ± 0.4% for linear kernel SVM. Multivariate methods often balance between intuitive results and good performance.^21^ The PCA/LDA has the advantage that the LD shows the component of best separation, and it is easier to relate the variance of specific spectral features and hence to relate it to the underlying biology. The simple DT and C5.0 output are of single bands, which is the simplest and most intuitive output to relate with the spectral changes from the ones reviewed here but also gives a less powerful classifier. More information about these models can be found in the Supporting Information.

#### 3.3.4 Average and multivariate analysis of results of multiple cell lines

**Figure 3:**
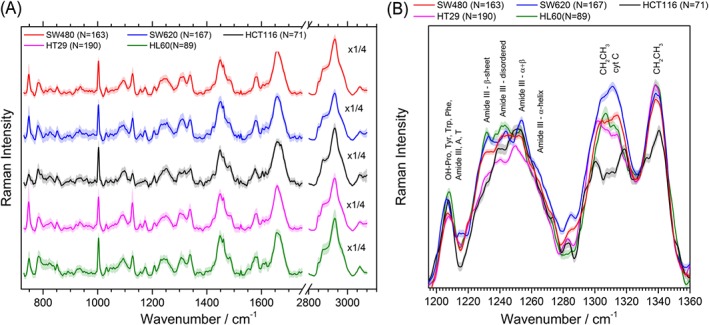
(a) Average single cell spectra of the different cell lines used, where the error shows one standard deviation. (b) Average of the spectra around the Amide III region with tentative assignment. The lighter coloured broad line represents the standard error [Colour figure can be viewed at http://wileyonlinelibrary.com]

Figure 3a shows the averaged spectra of each of the cell lines. The Amide III region is shown in Figure 3b.

The HL60 cell line shows lower intensity in the 749 cm^−1^ band but not in other cytochrome‐related bands, probably indicating lower DNA content than the adherent cell lines but with higher intensity in the 782 cm^−1^ band associated with nucleic acids, which could be showing a higher RNA content. When looking at the 782 cm^−1^ band and the 810 cm^−1^ bonded phosphates band, the normalized intensity follows the trend HL60 > HCT116 > SW620 > HT29 > SW480. Interestingly, the modal number of the cell lines according to the literature shows the inverse trend HT29 (68–72) > SW480 (58) > SW620 (50)^58^>HL60 (46) > HTC116 (45).^59^, ^60^ As the peaks are normalized to the Amide I, this could be showing that the protein content is strongly correlated with the DNA content.

Previous studies of xenographs of HT29, HCT116, and SW620 cells showed that the most common metabolites were amino acids and lactate,^61^ indicating that the 1,725 cm^−1^ peak associated with ν C=O and the 885 and 898 cm^−1^ peaks probably have a strong contribution from lactate. These peaks show the trend HCT116 > SW620 > HT29 > SW480 ≈ HL60, which also agrees with previous magnetic resonance spectroscopy results.^53^, ^61^ This can be attributed to the Warburg effect, due to which highly proliferative cancerous cells have increased lactate contents; HCT116, SW620, and HT29 are known to have lower doubling times than SW480 and HL60 cells.^51^, ^62^, ^63^, ^64^ For the carcinoma cell lines, the lactate contribution appears to be correlated with the cancer stage.

In general, HL60 have higher phosphate than the colorectal cell lines. For the colorectal cancer cell lines, differences between the 810 and 828 cm^−1^ peaks and the 1,095 cm^−1^ peak could be indicating that HCT116, HT29, and SW620 cells have more bonded phosphates than SW480 cells and that HCT116 cells have lower free phosphate concentration than the other cell lines.

Spectral regions around Amide III were fitted with Gaussian peaks as shown in Figure S3. The Amide III band has very different shapes for the different cell lines. Both HL60 and SW620 cells showed high contributions for both β‐sheet, disordered, and α + β secondary structure, with a lower contribution of α‐helix structure. In contrast, HCT116, HT29, and SW480 cells showed reduced β‐sheet peak height with higher disordered and α + β contributions, suggesting that increased ratio of α + β/β‐sheet could be a signature of primary colorectal cancer. This would merit further investigation. SW620 and SW480 cells showed higher α‐helix contribution than the other cell lines. Amide I fitting showed a similar trend to the one seen in Amide III within fitting error, where HCT116 showed a significant higher contribution of random coils. In terms of amino‐acids content, the phenylalanine peak had a lower contribution for the adenocarcinoma primary cell lines (HT29 and SW480) followed by SW620 cells, with higher contribution for the HCT116 cells and the HL60 cells.

Fitting to the CH stretching region, Figure S3 showed SW480, HL60, and HCT116 to have higher contributions above 2,930 cm^−1^. HCT116 cells had a very low contribution in the 2,848 cm^−1^ CH_2_ symmetric band compared with the other cells, showing higher fatty acids levels for HCT116 cells than for SW620 cells.^39^, ^61^ Although for the adenocarcinoma cell lines, the contributions above 2,900 cm^−1^ appear to be dependent on the cancer stage (SW480 > HT29 > SW620), a promising biomarker that would need to be confirmed in further experiments.

In summary, the results suggest that HL60 cells show low DNA, lactate, β‐sheet content, and high bonded phosphates, lipids, disordered, and α + β secondary protein structure, clearly separating it from the colorectal cell lines. HCT116 cells showed lower cytochrome C peaks, β‐sheet content, free phosphates, and CH_2_ symmetric stretching band, and higher lactate, disordered and α + β contributions, all possible signatures of colorectal carcinoma compared with adenocarcinoma. For the colorectal adenocarcinoma cell lines, the lactate contribution measured using the 1,725 cm^−1^ peak seems to be proportional to the cancer stage, whereas the CH stretching contributions above 2,900 cm^−1^ were inversely proportional to the cancer stage. This would indicate that more malignant cells would tend to increase their lactate/protein ratio—due to the Warburg effect—while decreasing their lipid/protein contents. Additionally, SW620 cells showed lower phenylalanine peak and lower α + β:β‐sheet ratio and SW480 showed lower bonded phosphates.

**Figure 4:**
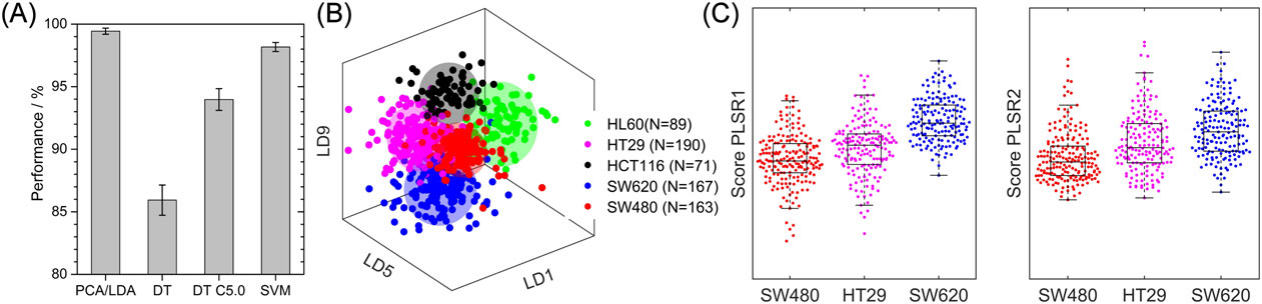
(a) Performance of the four classification methods when applied to the SW620 and SW480 datasets. (b) 3D plot of chosen LDs of the different cell lines, where the spheres are centred on the average values and have radius of two standard deviations. (c) Composite box plots/bee‐swarm plots for the scores of each cell line in the PLSR Components 1 and 2, showing a linear trend with disease stage. The *p* values for each pair was found to be <.001 [Colour figure can be viewed at http://wileyonlinelibrary.com]

In general, the differences between the cell lines are subtle when looking at the average spectra, but are clear when applying the PCA/LDA model. The LD model consisted of 10 LDs, each of them maximizing the separation between a pair of the five cell lines. Figure 4b shows a 3D plot of three selected LDs that showed the best separation where the average and two standard deviations of each cell population has been shown as a sphere. All cell lines show very clear clustering separated from each other. HL60 clusters further from the other cell lines in LD1 as the only nonadherent cell line. HCT116 shows clear separation with the other colorectal cell lines, underlying the ability of Raman spectroscopy to separate between different cancer types even within the same organ. SW480 and HT29 lie very close to each other and show the worst separation as expected given that they both originate from colorectal adenocarcinoma. The PCA/LDA model using a 10‐fold cross validation showed a performance of 92.4 ± 0.4%.

In order to find possible biomarkers for disease stage in colorectal adenocarcinoma, the spectra of the SW480/HT29/SW620 cell lines were fitted to a PLRS model looking for spectral features that change linearly with disease stage. Only Components 1 and 2 that showed a significant increase with cancer stage (*p* < .01) were considered for analysis, and the cell scores for each cell line are plotted in Figure 4c. Among other potential biomarkers noticed in the average analysis, results showed a decrease of the stretching peak at 2,850 cm^−1^, decrease of the DNA peak at 787 cm^−1^, increase of the 1,438 cm^−1^ peak with a decrease around (1,465–1,490 cm^−1^), and a decrease in the Amide I contribution above 1,675 cm^−1^. An increase at 810–813 cm^−1^ (bonded phosphates and phosphodiester) may be linked with increased phosphorylated status with cancer stage and/or increased nucleic acid content. The 1,556 cm^−1^ peak related with double bonds and previously reported to increase in SW620 cells compared with SW480 cells,^45^ showed increase with cancer stage when considering HT29 cells. PLSR analysis also showed blue shifting of the phenylalanine peak at 1,002 cm^−1^ and the 1,174 cm^−1^ peak from the cytochrome C series and red shifting of the 747 and the 1,227 cm^−1^ cytochrome C peaks with advancing adenocarcinoma stage. The shape of the components is shown in Figure S7.

## 4 CONCLUSIONS

We have shown that Raman spectroscopy of hundreds of live cells can readily be used to distinguish between different cell types and between different colorectal cancer cell lines including a primary and secondary cell line from the same patient.

For the metastatic model system, we found that when normalizing to the Amide I peak, secondary tumour cells (SW620) displayed higher saccharides, phosphates, nucleic acid content, α‐helix, β‐sheet, and α + β secondary structure, increased ratio of α:β secondary structure and increased ratio of CH_2_:CH_3_ stretching bands. The SW480 cells displayed a higher proportion of disordered structure and increased overall CH stretching intensity. PCA discrimination indicated that the cytochrome C peaks accounted for most of the within sample variability whilst the protein, nucleic acids, and lipid‐associated peaks gave the largest variability between cell lines.

Supervised multivariate methods like LDA/PCA and SVM results yielded >98% accuracy in classification between the SW620/SW480 cell lines compared with DTs and C5.0 DTs, that gave good but lower performance, though they allowed obtaining single peak biomarkers.

When comparing multiple colorectal cancer cell lines, we found that the primary colorectal cancer cell lines (SW480, HT29, and HCT116) showed increased α + β:β‐sheet ratio in the Amide III band compared with the HL60 and SW620 cells. The carcinoma cell line HCT116 showed lower cytochrome C, CH_2_ symmetric stretching and free phosphates, and higher lactate contributions compared with the adenocarcinoma cell lines. The analysis of the average and PLSR analysis with the colorectal adenocarcinoma stage showed an increase on the lactate contribution at 1,725 cm^−1^, the 810–813 cm^−1^ peak associated with bonded phosphates, and phosphodiester and the 1,556 cm^−1^ peak related with double bonds and a decrease on the contributions above 2,900 cm^−1^, the DNA peak at 787 cm^−1^, and the Amide I contribution above 1,675 cm^−1^ among others, and their possible applications as biomarkers deserve further study. Overall, the PCA/LDA performance for the separation of different cancer types was 92.4 ± 0.4% showing the potential of Raman spectroscopy to separate between live healthy and cancerous cells—in future, we seek to extend these studies to patient samples.

## CONFLICT OF INTEREST

The authors declare no competing financial and nonfinancial interests.

## Supporting information

Figure S1: Schematics of colorectal adenocarcinoma Duke stages A, B and C, showing the tumour advances through the bowel layer and the further invasion of the lymph node.
Figure S2: Phase contrast images of the SW480 cells and SW620 cells when grown in a flask. The SW480 cells show more epithelial morphology whilst the SW620 cells have a fibroblast‐like morphology (scale bar of 100 μm).
Figure S3: Averaged normalized Raman intensity for the CH‐stretching region, the Amide III and the Amide I, where the area around the curve corresponds to one standard deviation. (A1–5) CH‐stretching Raman intensity for each cell line fitted with 5 Gaussian peaks with tentative labelling. (B1–5) Amide III region fitted with 4 Gaussian peaks with tentative labelling. Peak position and width have been fixed between the cell lines.
Figure S4: (A) Scree plot showing the variance explained (left axis) and cumulative variance explained (right axis) of the first 25 PCs. (B) Performance of the LDA model when using 25 PCs and different numbers of cells in the training group, with a test group of 50 cells. The area shows the standard error and a Sigmoidal Weibull function was fitted to the data with a saturation value of 97.4 ± 0.3%.
Figure S5: histograms for the scores of components 1 to 4 for the SW620 and SW480 cells. PC1 and PC2 showed different average scores for each cell line, where PC2 showed the best separation. PC3 and PC4 showed similar average scores for each cell line, showing mainly within‐sample variability.
Figure S6: (A) Example of one of the trees fitted when using k‐fold validation to illustrate the classification process, with a performance of 83.3%. For each node, the cells whose intensities were lower than the value of the node are sent to the upper branch, and the ones higher, to the lower branch. Red symbolizes the SW480 and blue the SW620 cells, and the pie charts indicate the proportion of cells in each node. (B) Performance of a single tree when using different training set sizes (number of cells per cell line) and fitting it to the remaining cells. The error was estimated by performing 100 fits each with randomly chosen cells, and testing them in 50 of the remaining cells. (C) Bands chosen by the trees shown as vertical lines, where the line intensity is proportional to the frequency at which the bands were chosen. The averages of the two cell lines have been shown as a reference, and the area around the curves is two times the standard error (95% confidence interval). (D) 3D plot of the 3 most frequent bands obtained in the analysis of all of the fitted trees when using the C5.0 algorithm.
Figure S7: Shape of the two PLSR components that achieved separation between the SW480/HT29/SW620 populations with *p* < 0.01. Main peaks have been labelled.
Table S1: Main peaks observed in the averaged single cell spectra for SW480 and SW620 Cells^1–5^.
