# Supplementary material for: Biochemical fingerprint of colorectal cancer cell lines using label‐free live single‐cell Raman spectroscopy
Source: J Raman Spectrosc. 2018 May 2;49(8):1323–32. doi: 10.1002/jrs.5389 (PMC6473482; doi:10.1002/jrs.5389)
Supplement: Supplementary file 1 — Figure S1: Schematics of colorectal adenocarcinoma Duke stages A, B and C, showing the tumour advances through the bowel layer and the further invasion of the lymph node. Figure S2: Phase contrast images of the SW480 cells and SW620 cells when grown in a flask. The SW480 cells show more epithelial morphology whilst the SW620 cells have a fibroblast‐like morphology (scale bar of 100 μm). Figure S3: Averaged normalized Raman intensity for the CH‐stretching region, the Amide III and the Amide I, where the area around the curve corresponds to one standard deviation. (A1–5) CH‐stretching Raman intensity for each cell line fitted with 5 Gaussian peaks with tentative labelling. (B1–5) Amide III region fitted with 4 Gaussian peaks with tentative labelling. Peak position and width have been fixed between the cell lines. Figure S4: (A) Scree plot showing the variance explained (left axis) and cumulative variance explained (right axis) of the first 25 PCs. (B) Performance of the LDA model when using 25 PCs and different numbers of cells in the training group, with a test group of 50 cells. The area shows the standard error and a Sigmoidal Weibull function was fitted to the data with a saturation value of 97.4 ± 0.3%. Figure S5: histograms for the scores of components 1 to 4 for the SW620 and SW480 cells. PC1 and PC2 showed different average scores for each cell line, where PC2 showed the best separation. PC3 and PC4 showed similar average scores for each cell line, showing mainly within‐sample variability. Figure S6: (A) Example of one of the trees fitted when using k‐fold validation to illustrate the classification process, with a performance of 83.3%. For each node, the cells whose intensities were lower than the value of the node are sent to the upper branch, and the ones higher, to the lower branch. Red symbolizes the SW480 and blue the SW620 cells, and the pie charts indicate the proportion of cells in each node. (B) Performance of a single tree when using different train [file JRS-49-1323-s001.docx]

Supporting Information: Biochemical fingerprint of Colorectal Cancer cell lines using label-free live single-cell Raman spectroscopy

Julia Gala de Pablo^a^, Fern J. Armistead^a^, Sally A. Peyman^a^, David Bonthron^b^, Michael Lones^c^, Stephen Smith^d^, Stephen D. Evans^a^


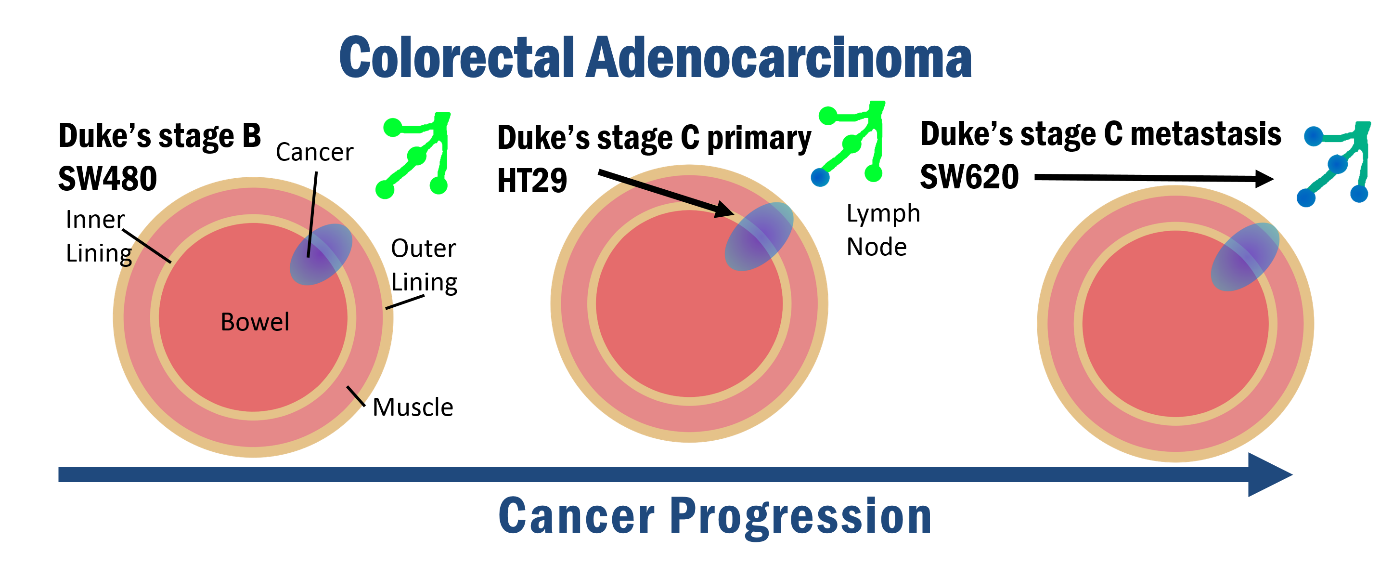


**Figure S1**: Schematics of colorectal adenocarcinoma Duke stages B, C (primary) and C (metastasis), showing the tumour advances through the bowel layer and the further invasion of the lymph node.


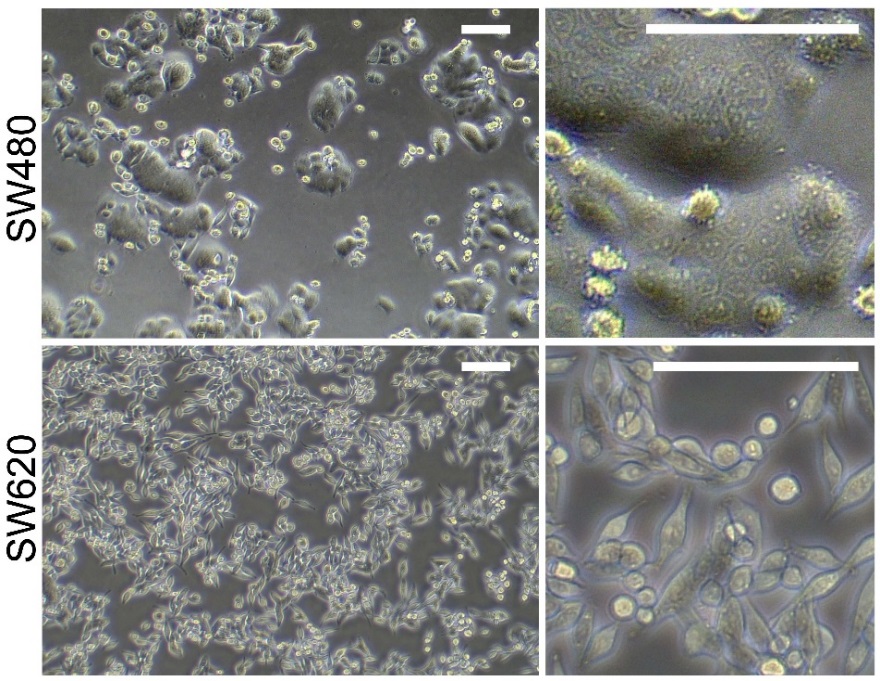


**Figure S2**: Phase contrast images of the SW480 cells and SW620 cells when grown in a flask. The SW480 cells show more epithelial morphology whilst the SW620 cells have a fibroblast-like morphology (scale bar of 100 µm).

| **Peak position (cm^-1^)** | **Peak position and assignment** | **Peak position (cm^-1^)** | **Peak position and assignment** |
| --- | --- | --- | --- |
| 747 | DNA, Cytochrome C Resonant Raman | 1339 | Tryptophan, CH_2_/CH_3_ wagging and twisting modes, C-C stretch of phenyl |
| 782 | U, T, C (ring breathing modes in the DNA/Nucleic acid bases) | 1448 | CH_2_ bending, C-H deformation |
| 1003 | Phenylalanine | 1461 | CH_2_/CH_3_ deformation  CH_2_, Disaccharides, sucrose |
| 1095 | Lipids, C-N and O-P-O of Nucleic acids | 1585 | Cytochrome C Resonant Raman |
| 1128 | Cytochrome C Resonant Raman | 1653-1674 | Amide I |
| 1156 | C-H vibrations | 1657 | Triglycerides |
| 1175 | C-C vibrations | 2848 | CH2 νs |
| 1207 | Hydroxyproline, Tyrosine  Tryptophan, Phenylalanine, Adenine, Thymine ring breathing modes, Amide III | 2873 | CH_3_ νs (FR) |
| 1230 | Amide III - β-sheet | 2901 | CH_2_ νas |
| 1243 | Amide III - disordered | 2934 | CH_3_ νs |
| 1254 | Amide III - α+β | 2973 | CH_3_ νas |
| 1263 | Amide III - α-helix | 3065 | -CH asymmetric stretching |
| 1311 | Cytochrome C Resonant Raman |  |  |

**Table S1**: Main peaks observed in the averaged single cell spectra for SW480 and SW620 Cells^1–5^.

**Figure S3**: Averaged normalized Raman intensity for the CH-stretching region, the Amide III and the Amide I, where the area around the curve corresponds to one standard deviation. (A1-5) CH-stretching Raman intensity for each cell line fitted with 5 Gaussian peaks with tentative labelling. (B1-5) Amide III region fitted with 4 Gaussian peaks with tentative labelling. Peak position and width have been fixed between the cell lines.


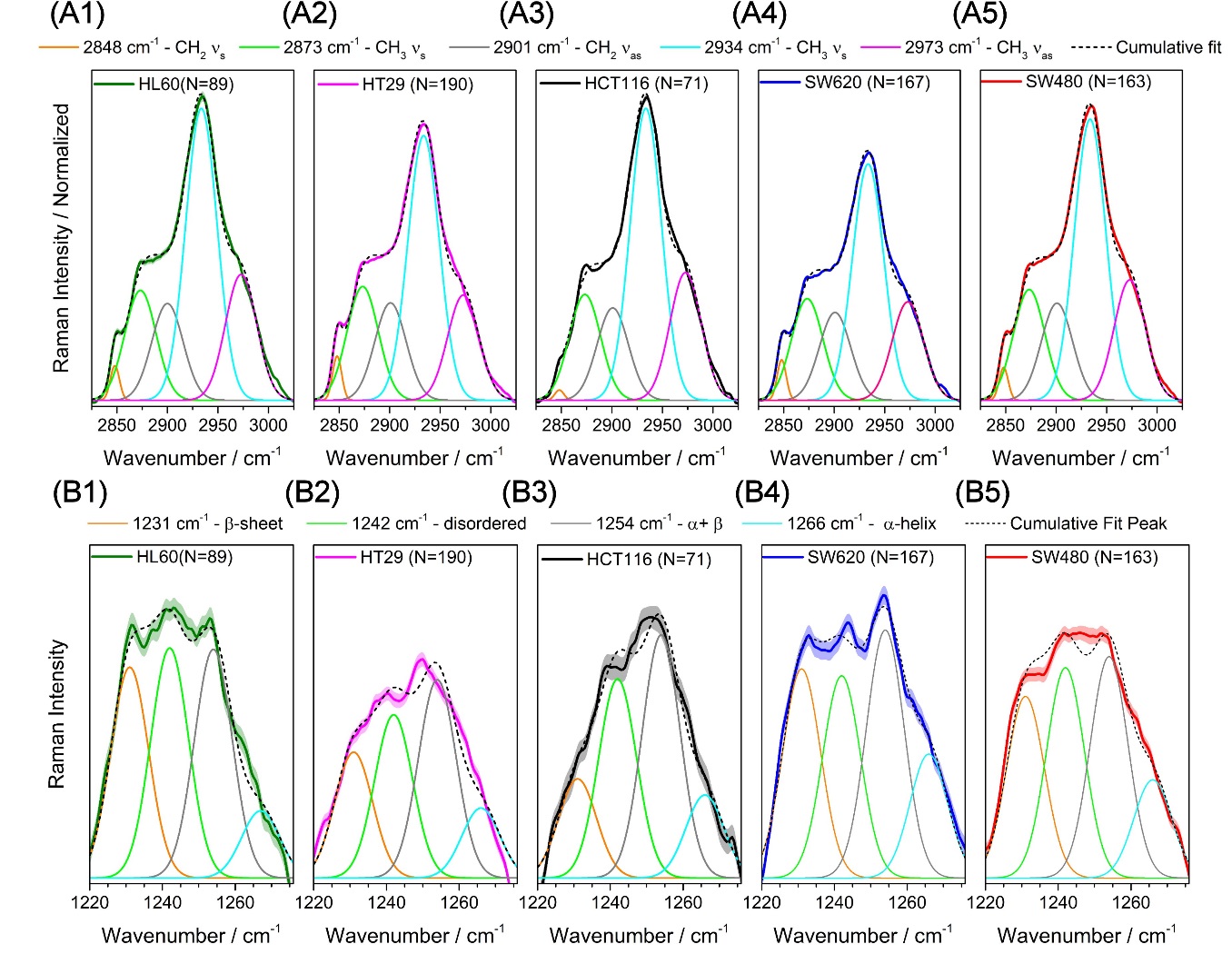


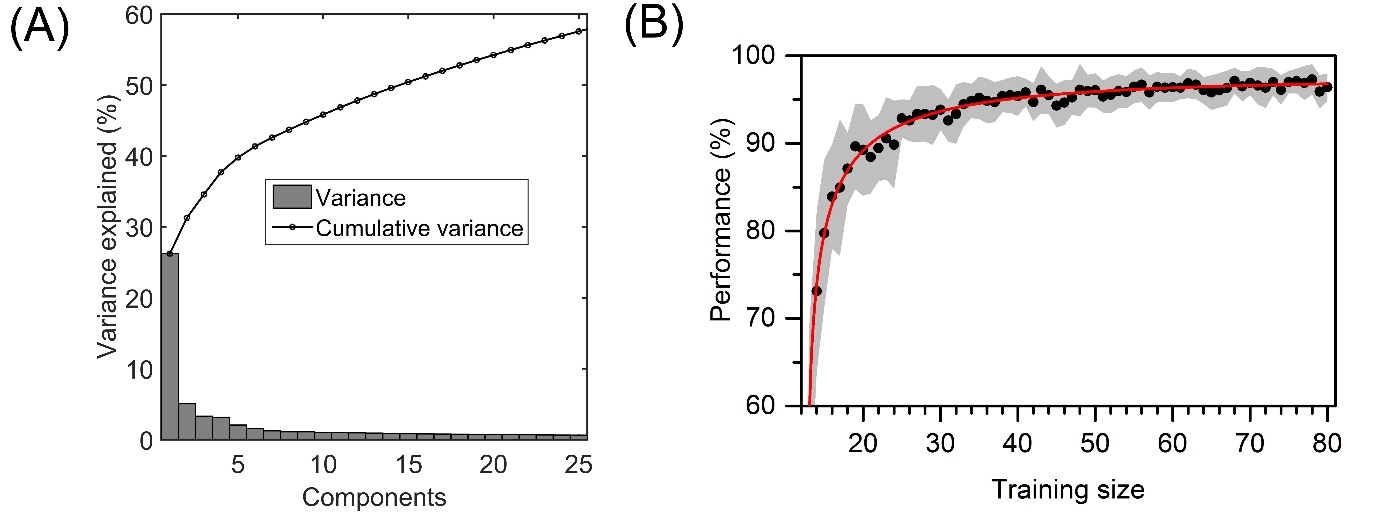


**Figure S4**: (A) Scree plot showing the variance explained (left axis) and cumulative variance explained (right axis) of the first 25 PCs. (B) Performance of the LDA model when using 25 PCs and different numbers of cells in the training group, with a test group of 50 cells. The area shows the standard error and a Sigmoidal Weibull function was fitted to the data with a saturation value of 97.4±0.3%.


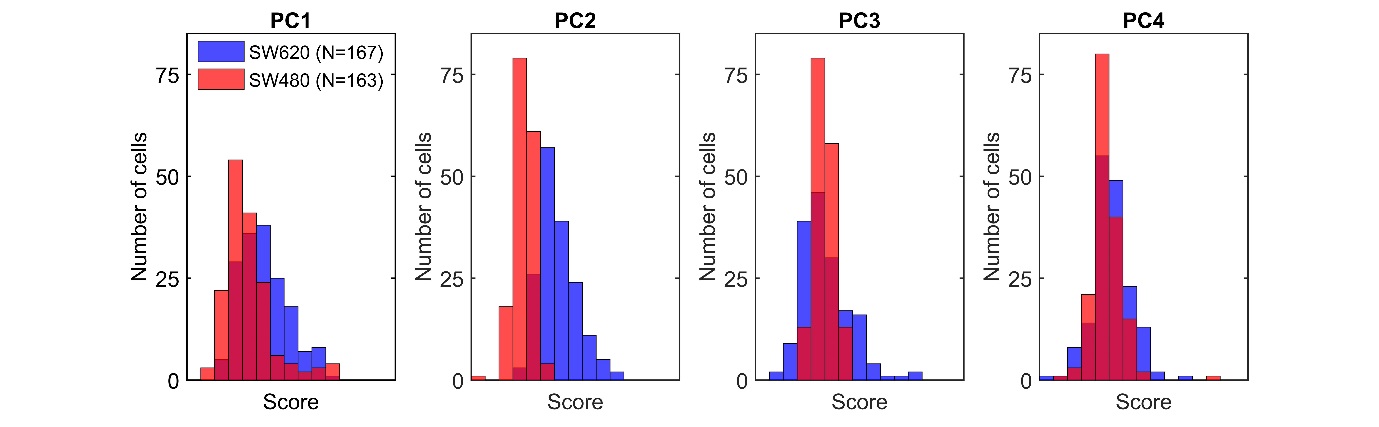


**Figure S5**: histograms for the scores of components 1 to 4 for the SW620 and SW480 cells. PC1 and PC2 showed different average scores for each cell line, where PC2 showed the best separation. PC3 and PC4 showed similar average scores for each cell line, showing mainly within-sample variability.

Supervised Multivariate Analysis: Discrimination Trees. A binary classification tree is based on choosing the best variables to classify the data by using multiple binary decisions. An example of this tree when using k-fold validation (k=10) is shown in S6A, where 90% of the 330 cell spectra available were used for the training (145 SW620 and 152 SW480 cells). The cells were classified by using 10 decisions. To avoid overfitting of the model, the test set was used to assess the performance. To choose the best size for the training dataset, trees were fitted to random training subsets of different sizes, and the performance was calculated by applying each of the models obtained onto a random testing subset of 50 cells from the testing set (see Figure S6B). When fitting a model with 5 repetitions of k-fold validation the average performance obtained was of 86±1%. It was common that lipid related peaks such as 2938 or 1455 cm^-1^ were chosen. The *peaks* chosen by any given tree varied slightly for different trees depending on the training subset. Thus using an algorithm for optimization of the trees by fitting multiple trees is advantageous.

Supervised Multivariate Analysis: C5.0 model. The data was fitted to a C5.0 model, where multiple trees are used to optimize the final tree used for classification. Figure S6C shows the bands most frequently chosen by the models as vertical lines, where the boldness of the lines is proportional to their frequency of appearance in the models, and the average spectra of the cells have been included as a reference. It can be seen that the Amide I region and the CH stretch regions are often chosen as classifiers. The band at 1455 cm^-1^ was chosen 99.1% of the time (CH_2_ assignments) whilst that at 1423 cm^-1^ (N-H in plane deformation) was chosen 93.9%. The second more commonly used band is the one at 2937 cm^-1^ (98.8%, CH_2_ asymmetric and CH_3_ symmetric stretching) surrounded by other frequent bands all chosen by more than 80% of the trees. This underlines the importance of the CH_3_ contributions for the discrimination between the cell lines. Interestingly, the 890 cm^-1^ peak is chosen 87.6% of the time (often labelled as saccharide band of methylene group). Proline and hydroxyproline related bands were also often chosen (933 cm^-1^ or 916 cm^-1^). Amide I bands are chosen less often (<75%). The intensities of the three most frequent bands were 3D plotted in Figure S6D.


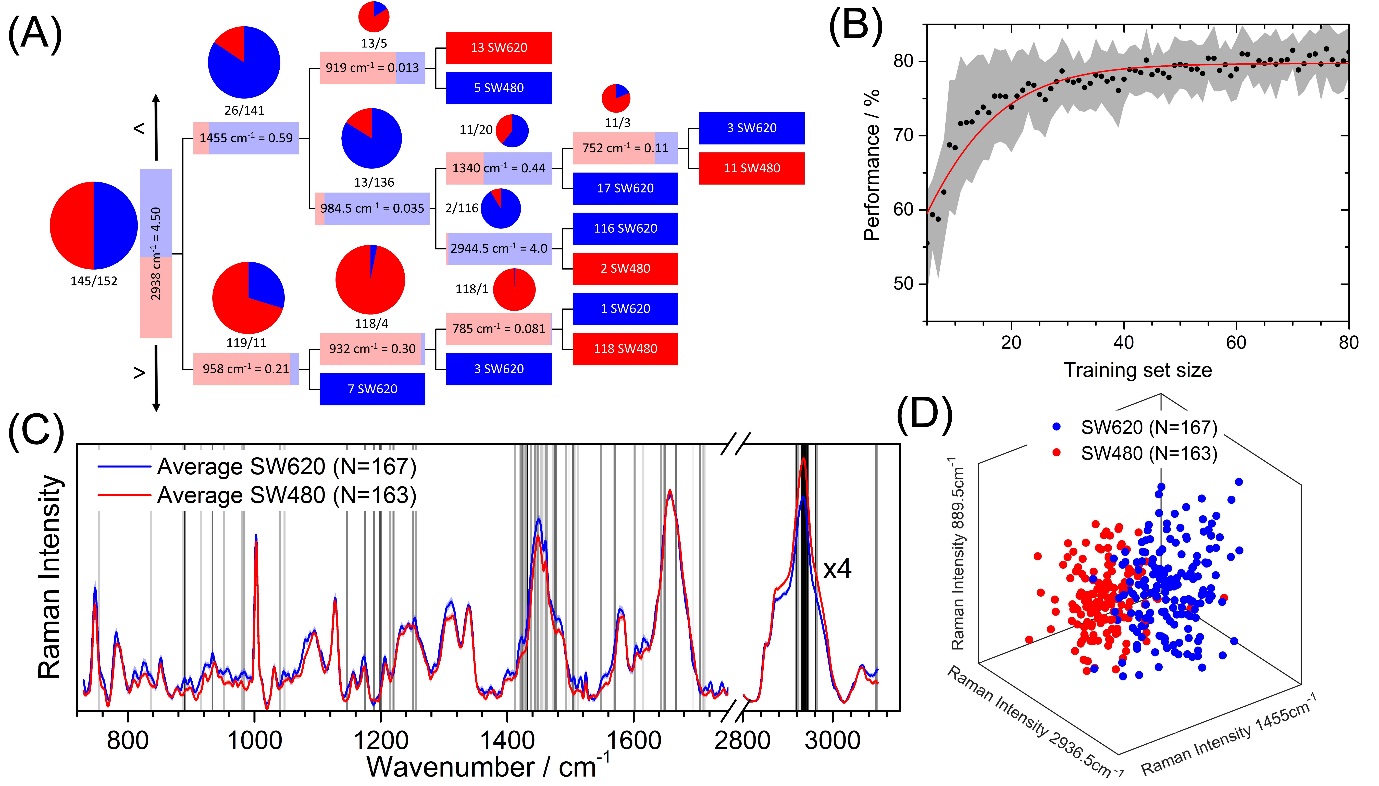


**Figure S6**: (A) Example of one of the trees fitted when using k-fold validation to illustrate the classification process, with a performance of 83.3%. For each node, the cells whose intensities were lower than the value of the node are sent to the upper branch, and the ones higher, to the lower branch. Red symbolizes the SW480 and blue the SW620 cells, and the pie charts indicate the proportion of cells in each node. (B) Performance of a single tree when using different training set sizes (number of cells per cell line) and fitting it to the remaining cells. The error was estimated by performing 100 fits each with randomly chosen cells, and testing them in 50 of the remaining cells. (C) Bands chosen by the trees shown as vertical lines, where the line intensity is proportional to the frequency at which the bands were chosen. The averages of the two cell lines have been shown as a reference, and the area around the curves is two times the standard error (95% confidence interval). (D) 3D plot of the 3 most frequent bands obtained in the analysis of all of the fitted trees when using the C5.0 algorithm.

Supervised Multivariate Analysis: SVM. Whilst DT models are relatively interpretable, they are often outperformed by other machine learning methods. A prominent disadvantage of SVMs, however, is that it is difficult to extract useful knowledge from the trained models. In our experiments, SVMs (linear kernel) achieved a significantly higher accuracy than both the DT and the DT C5.0 of 98.2±0.4%. The choice of kernel made no significant difference to the accuracy, suggesting that the problem is linearly separable, a conclusion that is also supported by the high accuracies achieved using LDA.


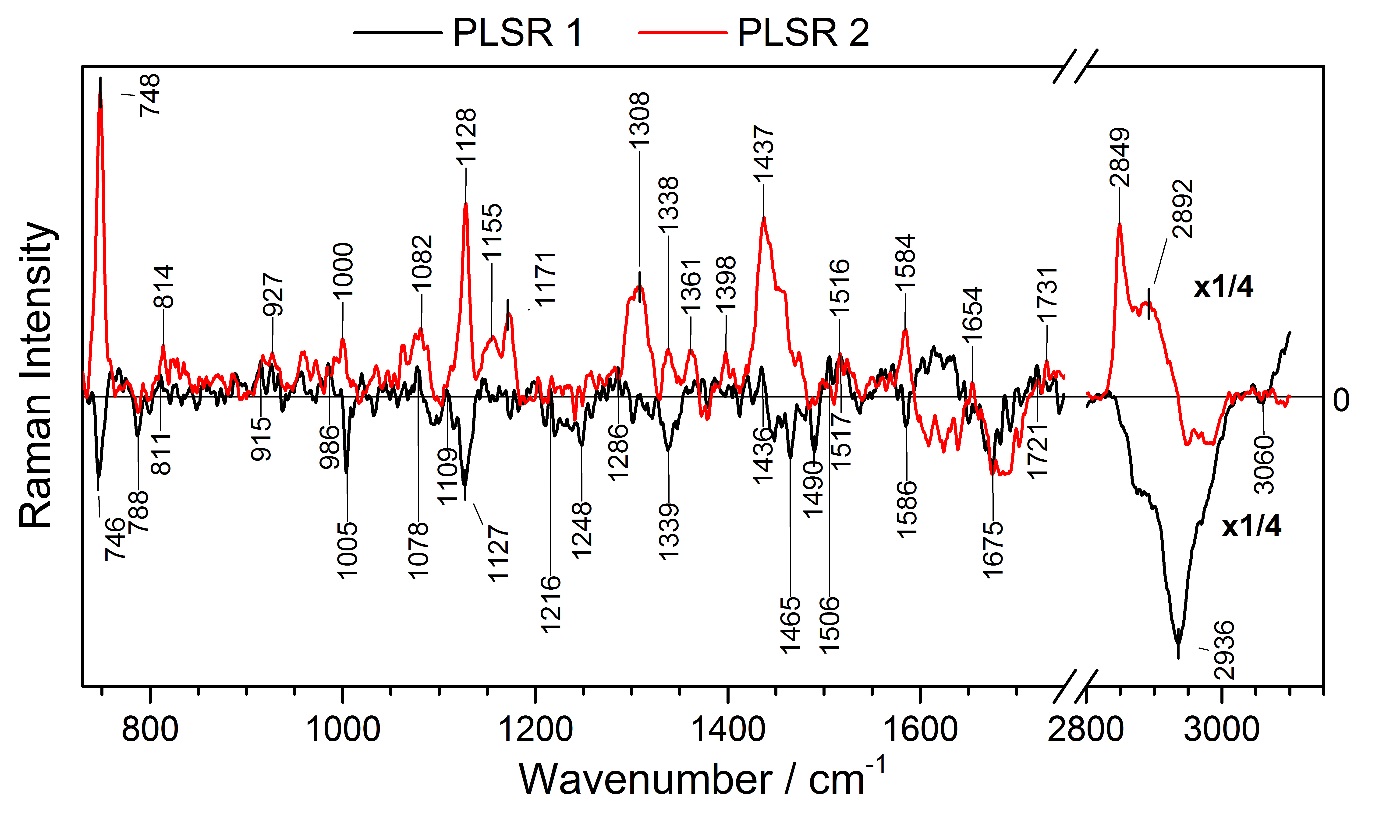


**Figure S7**: Shape of the two PLSR components that achieved separation between the SW480/HT29/SW620 populations with p<0.01. Main peaks have been labelled.
